# A New Perspective on the Status of the Intestinal Parasitic Infections in the Rural Areas of Fars Province South of Iran

**Published:** 2019-08

**Authors:** Mojtaba NOWROZI, Gholam Reza MOWLAVI, Mostafa ALISHAVANDI, Gholamreza HATAM

**Affiliations:** 1. Department of Parasitology and Mycology, School of Medicine, Shiraz University of Medical Sciences, Shiraz, Iran; 2. Department of Parasitology and Mycology, School of Public Health, Tehran University of Medical Sciences, Tehran, Iran; 3. Basic Sciences in Infectious Diseases Research Center, School of Medicine, Shiraz University of Medical Sciences, Shiraz, Iran

**Keywords:** Influential factors, Intestinal parasitic infection, Iran

## Abstract

**Background:**

Parasitoses are among the most important problems of most countries especially developing countries. We aimed to detect the situation of intestinal parasitic infections in the Farashband district in Fars Province South of Iran and identify influential factors in the escalation of parasitic diseases and to reduce them.

**Methods:**

Overall, 1009 participants from the age of 6 months to 90 years were selected from 3 cities and 15 villages of Farashband district, Fars Province South of Iran from 2015 to 2016. Parasitological methods such as the direct assay method, formalin-ether concentration method, and zinc sulfate flotation were used for diagnosis of worm eggs, cysts, and protozoa trophozoite. Susceptible and protozoan positive samples were stained using the Trichrome staining method. The modified acid-fast staining procedure was conducted for diarrheal samples and the results were used for diagnosis of coccidia.

**Results:**

Overall, 313 subjects were infected with at least one intestinal parasite (pathogenic and nonpathogenic). Helminthes infection and protozoan infection were observed in 9 (0.9%) and 304 (30.13%) participants, respectively. Fecal samples of 34 patients with diarrheal feces were used to prepare smears for further examinations using the Ziehl-Neelsen staining method. Examinations showed no infection with coccidia.

**Conclusion:**

Helminthes infection has decreased drastically but protozoan infection is still considered a health issue in this region. It is possible to reduce parasitic infections through proper measures such as increasing public awareness and education the public, especially children on health problems with education courses.

## Introduction

Parasitoses are among the most important problems of most countries, especially developing countries (1). The high prevalence of these diseases is attributed to poor hygiene, poverty, lack of education on health, and contaminations of soil, water and food (2). In fact, infection and contamination with intestinal parasites are health problems associated with factors such as personal behavior, social relations, and society’s cultural/economic status. On the other hand, one of the criteria for assessing health and hygiene levels, especially in tropical regions and third-world countries, is the frequency of parasitic infections (3, 4).

Intestinal protozoan or helminthes parasites are commonly found in the tropical and subtropical regions*. Ascaris lumbricoides, Entamoeba histolytica, E. dispar,* hookworms*,* and *Trichuris trichiura* are among the most prevalent parasites in the world (5). The prevalence of hookworms is less than 0.1% in Iran (6) and that of *E. coli, Endolimax nana,* and *Hymenolepis nana* is also 1.38%, 0.92%, and 0.19%, respectively (7). Among Khuzestan nomads, the prevalence of *Giardia lamblia*, *E. coli*, and *Blastocystis hominis* has been 10.91%, 9.37%, and 2.4%, respectively. The most common intestinal worms are *H. nana* (2.54%), *Strongiloides stercoralis* (0.6%), and *Trichostrongylus* spp. (0.47%) (1). A study on 1525 samples in rural and urban areas of Islamshahr town south of Tehran the capital of Iran, reported the following frequencies for helminth infections: *H. nana* 1.4%, *A. lumbricoides* 0.3%, *Dicrocoelium dendriticum* 0.1%, *Taenia solium* 0.2%, *Trichostrongylus* spp. 0.1%, *S. stercoralis* 0.3%, and *T. trichiura* 0.1% (8). In Iran, the prevalence of intestinal parasites was as follows: *G. lamblia* 10.9%, *A. lumbricoides* 1.5%, *E. histolytica* 1%, and *Enterobius vermicularis* 0.5%. The highest prevalence of infection was seen in the 2–14-year (25.5%) age group among rural residents (23.7%) (9). Development of proper health plans for controlling and fighting a disease in each region calls for precise and novel epidemiologic information. To this end, analysis of intestinal parasitic infections results in an understanding of environmental factors and the prevalence of infections and can be used as an effective health measure. Since no comprehensive and purposeful research had been conducted on intestinal parasitic infections in Farashband district, we aimed to carry out the present research given the importance of the physical and mental health of residents and appropriate health decisions.

## Materials and Methods

This cross-sectional study was carried out from 2015 to 2016 in Farashband district, located in Fars Province, south of Iran. It neighbors Lar town in the south and Qir, Karzin, and Firuzabad towns in the east. It is located 170 km from Shiraz city the capital of Fars Province. This region has arid and hot weather, and its average annual temperature is over 25 °C. Its annual precipitation is approximately 250 to 300 mm and its altitude is 800 m. In 2011 census, the population of the urban and rural parts of this town was 42670 and 15366, respectively.

This information was obtained after the people completed the consent form for participation in the project.

Of the study participants, 559 (55.4%) were urban and 450 (44.6%) were rural residents. Moreover, 506 (50.1%) were male and 503 (49.9%) were female. The age range of the participants was 6 months to 90 years (Table 1).

**Table 1:** Towns and villages of Farashband district and number of samples collected from each

***Code***	***Study town and village***	***Sample size and percentage (%)***
1	Farashband town	314 (31.11)
2	Dahrom town	151 (14.9)
3	Nojin town	94 (9.31)
4	Pahnapahn Village	61 (6.05)
5	Kenar Malek Village	13 (1.29)
6	Balut Abad Village	47 (4.66)
7	Aviz Village	39 (3.86)
8	Khoshab Village	34 (3.37)
9	Khormayek Village	8 (0.79)
10	Hussein Abad Village	12 (1.19)
11	Chah Sholi Village	29 (2.87)
12	Qanat Baq Village	12 (1.19)
13	Dezhgah Village	34 (3.37)
14	Goori Village	66 (6.54)
15	Shahid Village	23 (2.28)
16	Khan Yek Village	17 (1.68)
17	Savar Qeyb Village	25 (2.47)
18	Ahmad Abad Village	30 (2.97)
	Total	1009

Overall, 1009 fecal samples were randomly collected from Farashband, Dehram, and Nojin towns and 15 villages in the north, south, east, west, and center of this region. The number of samples was determined (N =1000) based on the opinion of a statistician using Power SSC. After making arrangements with Farashband town Health Center and the health house of each village and informing people and social workers, measures were taken to collect the fecal samples. By visiting them in their residence, information was provided to them on intestinal parasites and they were encouraged to cooperate in the collection of fecal samples.

Totally, 1009 participants from the age of 6 months to 90 years were selected from 3 cities and 15 villages of Farashband town Demographic and personal information of the participants was obtained using an informed consent completed form by each participant.

Parasitological methods such as the direct assay method, formalin-ether concentration method, and zinc sulfate flotation were used for diagnosis of worm eggs, cysts, and protozoa trophozoite. Susceptible and protozoan positive samples were stained using the Trichrome staining method. The modified acid-fast staining procedure was conducted for diarrheal samples and the results were used for diagnosis of coccidia. Statistical analyses and statistical tests such as the chi-square test revealed that factors such as residence (rural or urban) and lack/presence of health centers in residential areas are among the factors influencing the prevalence of parasitic infections in this area.

## Results

Of the study participants, 559 (55.4%) were urban and 450 (44.6%) were rural residents. Moreover, 506 (50.1%) of them were male. The participants’ age range was 6 months to 90 years (Details are available in Tables 2 and 3).

**Table 2:** Prevalence of intestinal parasitic infections according to species (N = 1009)

***Parasite***	***No***	***Infected %***
*Hymenolepis nana*	5	0.49
*Enterobius vermicularis*	4	0.39
*Giardia duodenalis*	99	9.81
*Entamoeba histolytica/dispar*	13	1.28
*Entamoeba coli*	121	11.99
*Endolimax nana*	9	0.89
*Iodamoeba butschlii*	1	0.1
*Chilomastix mesnili*	28	2.77
*Blastocystis hominis*	33	3.27
Total	313	0.31

**Table 3:** Factors associated with protozoan infections among patients (N: 307)

***Characteristics***	***Scale***	***Total, N (%)***	***Infected, N (%)***	***P- value***
Age groups (yr)	>10	182(18.04)	56 (30.77)	0.488
	10 ≤	827 (81.96)	251 (30.35)	
Level of Education	Educated Not educated	667 (79.48) 342 (20.51)	200 (29.99) 107 (31.29)	0.361
Gender	Male	506 (50.14)	151 (29.84)	0.368
	Female	503 (49.85)	156 (31.02)	
Family size	≤ 7	977 (96.83)	299 (30.60)	0.322
	> 7	32 (3.17)	8 (0.25)	
Address	Urban	559 (55.40)		0.004
	Rural	450 (44.60)		
Health centers				0.005

## Discussion

Given the improved status of health in Iran, the prevalence of parasitic infections has decreased drastically in recent years. In addition to the improved health, other factors such as increased public awareness improved economic status of families, and use of healthy and treated drinking water have led to a decrease in parasitic infections, especially helminth infections in Iran. In Tonekabon villages (north of Iran), the frequency of infection with *T. trichiura*, *A. lumbricoides*, *S. stercoralis*, hookworms, *Trichostrongylus* spp., and *H. nana* was 22.5%, 16.3%, 10.3%, 4.6%, 3.7%, and 3%, respectively (10). In Abyek County (Qazvin), the highest was 13.1%, *G. lamblia* 3*%, E. coli* 2.8%, and *E. nana* 2.3% (11). A comparison of previous research results with the present study findings reflects a drastic decrease in pathogenic parasites, especially helminth infections. Table 3 displays the relationship between personal information and parasitic infections. *P*-value is expressed based on results of statistical analyses.

According to statistical studies and data analyses, the prevalence of parasitic infections in Farash-band town is significantly related to the living place (urban or rural) and presence of health and treatment centers near their places of residence. All social factors and personal behavior can contribute to development of parasitic infections. Analysis of the prevalence of parasitic infections in the urban and rural regions has been carried out in many studies and the present research shows higher prevalence of infection in rural areas (as compared to urban areas). The highest prevalence was observed in Pahnapahn, Dezhgah, and Balut Abad villages. Based on statistical analyses, there was a significant relationship between the residence of people and prevalence of parasitic infections (*P*<0.05). As to age and gender, the highest number of infection was among women (156 cases, 31.02%) and the highest prevalence was in the 21–40 years’ age group. The level of education can also highly contribute to development of parasitic infections. The highest prevalence was among the participants with primary school education. Profession (especially farming and dairy farming) can intensify parasitic diseases.

In Farashband region, there was no significant relationship between the profession and parasitic infections. Fortunately, all participants used healthy/treated drinking water. This result reflects the increase in public health status as compared to the past decades. On the other hand, the prevalence of infection decreased considerably. Parasitic infection was in no significant relationship with age and gender; this is in line with our finding (10, 11). The research done on parasitic infection showed no significant relationship with age, gender, profession, and education level (1). However, there was a significant relationship between helminth infection and region. The presence of health and treatment centers near the living place is another factor involved in the prevalence of these infections, given the shortage of health centers and long distance from hospitals. Villagers are also not willing to visit health centers after manifestation of symptoms of parasitoses, and parasites such as *E. vermicularis* are especially prevalent among children and affected families.

## Conclusion

Numerous social factors and personal behavior can influence the prevalence of diseases and public health. A number of factors were studied and measures were taken to inform people. It is possible to effectively reduce parasitic diseases by informing people through social media, educating people especially children, constructing health centers in villages, and replacing animal and human fertilizers with chemical fertilizers.

## Ethical considerations

Ethical issues (Including plagiarism, informed consent, misconduct, data fabrication and/or falsification, double publication and/or submission, redundancy, etc.) have been completely observed by the authors.
